# Evaluating Self-Healing Behaviour of Asphalt Binders Modified with Phase-Change Materials, Polymers and Recycled Glass Powder

**DOI:** 10.3390/polym15081934

**Published:** 2023-04-19

**Authors:** Haya Almutairi, Hassan Baaj

**Affiliations:** Department of Civil and Environmental Engineering, University of Waterloo, Waterloo, ON N2L 3G1, Canada

**Keywords:** phase-change materials, polymers, recycled glass powder, self-healing asphalt, linear amplitude sweep, pure linear amplitude sweep

## Abstract

The objective of this paper is to evaluate the fatigue resistance and self-healing properties of asphalt binders modified with different types of additives (Styrene-Butadiene-Styrene (SBS), Glass Powder (GP) and Phase-Change Materials blended with Glass Powder (GPCM)). Two base binders were used in this study: a PG 58-28 straight-run asphalt binder and a PG 70-28 Polymer modified with 3%SBS. Moreover, the GP was added to the two base binders at two different percentages of 3.5% and 5% by binder weight. However, the GPCM was added with two different percentages of 5% and 7% by binder weight. In this paper, the fatigue resistance and self-healing properties were evaluated using Linear Amplitude Sweep (LAS) test. Two different procedures were adopted. In the first procedure, the load was applied continuously until failure (without a rest period), whereas, in the second procedure, rest periods of 5 and 30 min were introduced. The obtained results of the experimental campaign were ranked based on three different categories: Linear Amplitude Sweep (LAS), Pure Linear Amplitude Sweep (PLAS) and modified Pure Linear Amplitude Sweep (PLASH). The addition of GPCM appears to positively impact the fatigue performance of both straight-run and polymer-modified asphalt binders. Furthermore, when a short rest period of 5 min was introduced, the use of GPCM did not appear to improve the healing potential. However, a better healing capacity was observed when the 30 min rest period was applied. Moreover, the addition of GP alone to the base binder was not beneficial in improving fatigue performance based on LAS and PLAS methods. However, there was a slight reduction in the fatigue performance based on the PLAS method. Finally, unlike the PG 58-28, the healing capacity of the GP 70-28 was negatively affected by the addition of the GP.

## 1. Introduction

Self-healing materials have the potential to partially or completely heal and restore their mechanical properties when damaged [1]. Asphalt is a self-healing material that delays the growth of micro-cracks during rest periods [2] and at elevated temperatures [3]. As a result, the pavement’s service life is extended [4], and greenhouse gas emissions, as well as maintenance costs, are reduced [5].

The healing mechanism in asphalt can be described in three stages [6]: the first stage is the surface approach, where the flow of bitumen and the consolidation of stresses occur. The second is the wetting stage, in which the low surface energy causes the two faces of the crack to join. Finally, the last stage is where a complete recovery of the mechanical properties of the asphalt pavement occurs as a result of the diffusion and randomisation of asphaltene structures. Stage 1 is the fastest among all the stages since only the stiffness is recovered. In addition, both the stiffness and strength can be improved during the wetting and final stages due to the restoration of the original mechanical properties of the material.

Several studies have been conducted to understand the self-healing mechanism of asphalt binders. Moreover, many researchers strive to develop laboratory protocols to evaluate the healing capability of asphalt binders [7]. Currently, binders’ healing capacity is mainly assessed through Time Sweep (TS) and Linear Amplitude Sweep (LAS) tests, which are conducted using a Dynamic Shear Rheometer (DSR). For instance, Yue et al. [8] performed the Linear Amplitude Sweep Healing (LASH) test to compare the healing capacity of different asphalt binders. They found that the LASH test was able to rank the materials based on their healing ability under different aging conditions. They also found that different parameters, such as the duration of the rest period, the damage level at which the rest period is introduced and the aging condition of the binder, affected the Healing percentage (%HS). However, they observed that the oxidative aging condition of asphalt binders had the most significant negative impact.

Due to the nature of the applied load to the asphalt pavement that involves rest periods, in which pavements are not constantly subjected to continuous loading, attention is being given to the self-healing behaviour of asphalt pavement. The rest periods can be as short as a few seconds or extended to hours or days. In a study conducted by Bazin and Saunier [9], an asphalt binder recovered 90% of its initial tensile strength after a three-day rest period. Therefore, it is crucial to evaluate the self-healing behaviour of asphalt pavements and its effects on pavements’ fatigue life and resistance.

Incorporating some additives such as Polymers, Phase-Change Materials (PCMs) and Glass Powder (GP) into asphalt binders is expected to improve the self-healing properties of asphalt pavements, thus enhancing their overall mechanical properties. For instance, the use of PCMs can be associated with the self-healing process of asphalt mixtures because the latter is time-temperature dependent. It was found that high temperatures can enhance the healing properties during the recovery period [3]. The addition of PCMs into asphalt mixtures can produce temperature-control pavements, i.e., the pavements can possess the ability to adjust their temperature by storing and releasing thermal energy during the phase change process [10].

Moreover, the self-healing capability of polymer-modified bituminous materials was investigated by some researchers. However, limited findings were obtained on the effect of the addition of polymer on the healing process of the modified binders. For instance, Lee et al. [11] studied the healing behaviour of asphalt mixtures with different modifiers. They incorporated SBS, Styrene-Butadiene Rubber (SBR) and Gilsonite (GIL) into the asphalt mixes. The results showed a noticeable enhancement in the fatigue, rutting and healing performance of the asphalt mixtures with the addition of SBS compared to SBR and GIL.

However, a study by Kim, B. and Roque [12] observed a relatively small improvement in the healing rate of asphalt mixtures modified with SBS. Therefore, this paper aims to evaluate the fatigue characteristics of asphalt binders modified with different additives (Glass Powder (GP), Styrene-Butadiene-Styrene (SBS) and Glass Powder mixed with Phase-Change Materials (GPCM)) using the LAS test. Additionally, this study aims to investigate the healing properties of these binders at different rest periods using the PLASH test. This study is the first study that addresses the evaluation of the self-healing capacity of GP and GPCM. The paper presents a relative ranking of all binders tested based on their fatigue and self-healing capacity.

## 2. Materials Selection

### 2.1. Binder Properties

Two different binders were used in this study; the performance grade of the base asphalt binder was PG 58-28. However, the modified asphalt binder used was 3% Styrene-Butadiene-Styrene (SBS), and the performance grade for this binder was PG70-28. Table 1 shows the properties of the PG 58-28 and PG 70-28 asphalt binders.

### 2.2. Additives Properties

#### 2.2.1. Phase Change Materials (PCM)

**Figure 1:**
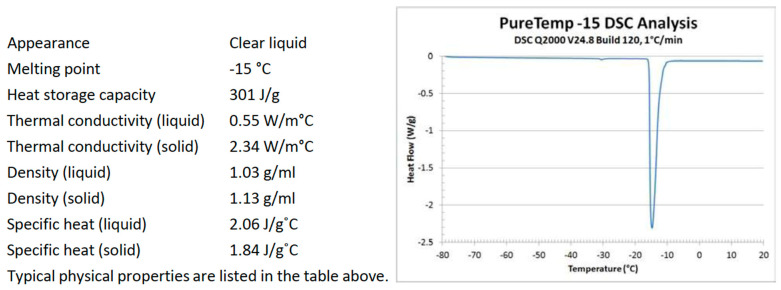
PureTemp −15 Technical Information. Reprinted from reference [16].

The PCM used in this study is an organic material derived from animal fats and plant oil. The used PCM falls into the organic category. This category classifies non-toxic, chemically stable and environmentally friendly materials [14]. The PCM used in this study possesses a good absorption capacity when used for thermal energy storage in buildings [15]. As thermal cracking is expected to occur in the asphalt layer when pavement temperature drops below 0 °C, the peak melting point temperature of the used PCM is −15 °C, as shown in Figure 1. The figure also shows the physical properties of the PCM, as provided by the material supplier (PureTemp^®^ Thermal Energy Storage Materials, Minneapolis, MN, USA).

#### 2.2.2. Recycled Glass Powder (GP)

**Figure 2:**
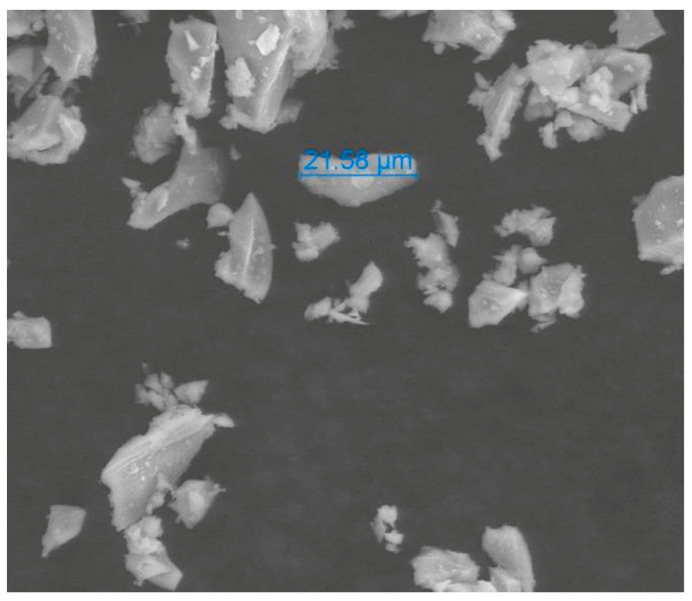
Particle Sizes and Shapes of GP.

The crushed glass obtained from household waste has become a major environmental problem. The collected glass consists of broken glass bottles and other containers of clear and coloured glass that cannot be recycled due to the difficulty of sorting it according to its type and colour in the recycling facility [17]. In this study, GP was collected from household waste in the province of Quebec, Canada. The particle sizes and shapes of GP were investigated using an Environmental Scanning Electron Microscope (ESEM) (FEI Quanta 250 FEG) at a low vacuum mode with the Energy Dispersive Spectrometer (EDS) removed. As shown in Figure 2, GP has irregular, flaky and angular particle shapes. It is also shown that GP has random and irregular pore structures. Moreover, the GP size was measured to be less than 25 µm.

#### 2.2.3. GP + PCM (GPCM)

**Figure 3:**
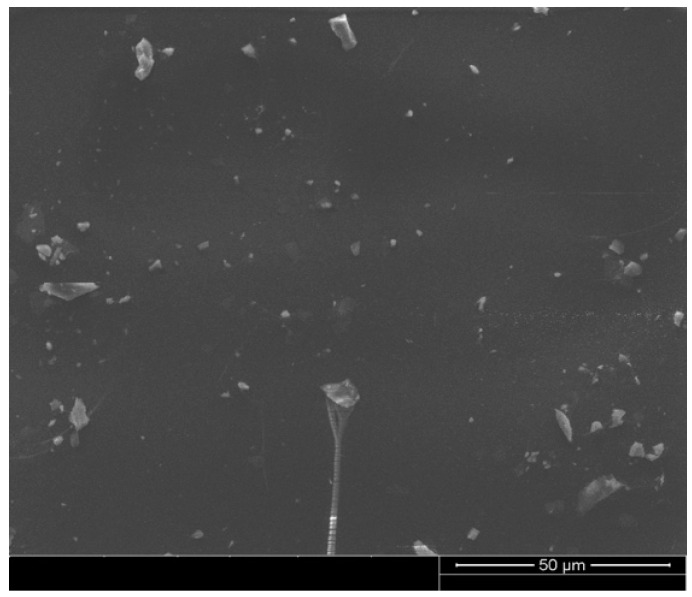
Microstructure of GPCM under ESEM.

Several studies investigated the direct blending approach of PCMs to asphalt binders and found that PCMs can easily leak from asphalt blends [18]. There is also a concern about the durability of the asphalt pavement modified with PCMs [19]. On the other hand, the use of GP as a filler is expected to contribute to the high-temperature stability of the binder at high temperatures. The physical characteristics of the glass powder, particularly the amorphous nature of the surface and the friction it creates, would contribute significantly to the stability of the mastic. They would compensate for the loss of stiffness caused by the PCM. GPCM’s observation using ESEM found that the GP particles are well dispersed in the mastic, as shown in Figure 3.

## 3. Experimental Program

### 3.1. Mix Design

**Figure 4:**
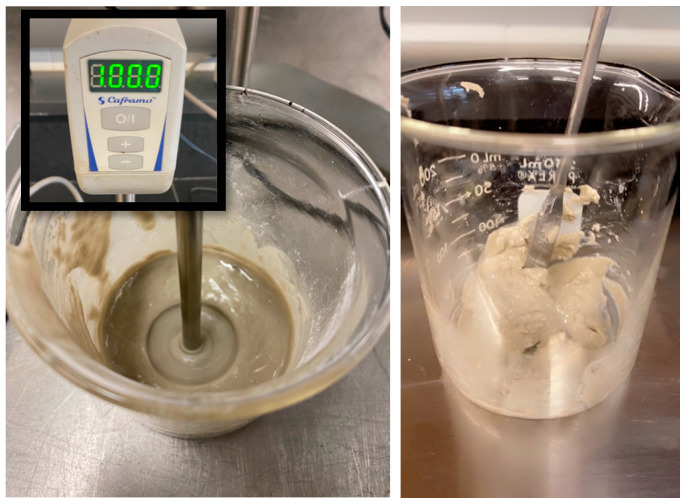
GPCM Preparation.

To investigate the self-healing and fatigue resistance properties of asphalt binders, 90 specimens in total were prepared and tested using DSR. Sixty and thirty specimens were prepared to evaluate the self-healing properties and the fatigue resistance, respectively. The GPCM paste was prepared by mixing GP with the PCM using a rotational mixer at 1000 rph, as shown in Figure 4. The mixing was conducted at room temperature for 2–3 min at a ratio of 70%GP and 30%PCM. The ratio/time was chosen to obtain a homogenous mix. When selecting the mixing time and temperature of an asphalt binder modified with GPCM, two main factors were considered: the nature of the additive and the degradation temperature obtained using a Thermogravimetric Analyzer (TGA). According to the TGA observations, the initial degradation temperature of the GPCM was relatively low compared to the base binders (PG58-28 and PG70-28). As a result, the mixing temperature for the control binder with the GPCM should not exceed 110 °C. On the other hand, the mixing temperature for the PG70-28 binder was chosen to not exceed 150 °C, as the latter requires a higher mixing temperature in order to obtain the required viscosity for mixing. It should be noted that approximately 2.5% mass loss in the GPCM was observed when the mixing temperature reached 150 °C.

**Figure 5:**
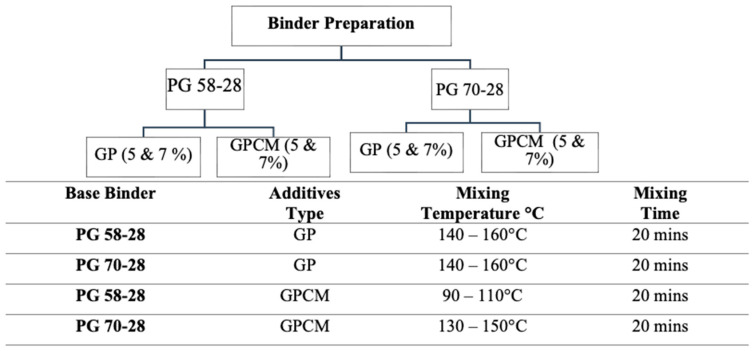
Schematic Flow Chart of Asphalt Binder Preparation.

The asphalt binder was modified with SBS, GP and GPCM at different percentages. The percentages, mixing temperature and time are shown in Figure 5. The literature suggested that the optimal addition dosage for glass powder could be up to 10% by binder weight [20,21,22]. On the other hand, no data can be found in the literature regarding the recommended amount of PCM that can be added to asphalt binders. As a result, two percentages (5 and 7%) were selected for this study. Table 2 shows a list of binders and mixtures used in this study.

### 3.2. LAS Test

Hintz and Bahia [23] introduced a LAS test to evaluate the fatigue performance of asphalt binders. The LAS test is a time-saving test that can substitute the time sweep (TS) test associated with uncertainty in the test duration required to complete the fatigue test [23]. The fatigue resistance of asphalt binders, which is presented in Table 2, was evaluated using the LAS tests in accordance with the AASHTO TP101-14. The test was performed using a Dynamic Shear Rheometer (DSR) with an 8 mm parallel plate geometry and a 2 mm gap setting at 20 °C. To quantify the damage resistance for asphalt binders, the samples were first subjected to a linear viscoelastic frequency sweep test at a constant temperature of 20 °C with a small shear strain. Then, an oscillatory strain sweep was applied to the same sample, with strain amplitudes linearly ranging from 0.1% to 30% at 10 Hz and the same temperature as used in the frequency sweep test.

Two approaches were adopted to investigate the damage characteristic of asphalt binders. The first one was the AASHTO TP 101 standard, which was used by Bahia (2013). This approach utilises the viscoelastic continuum damage mechanics (VECD) model to determine the fatigue life of the asphalt binder. The binder fatigue life N_f_ was calculated using Equation (1):

N_f_ = A (γ_max_)^−B^ (1)

where: γ_max_ is the maximum expected binder strain for a given pavement structure; A and B are regression coefficients.

**Figure 6:**
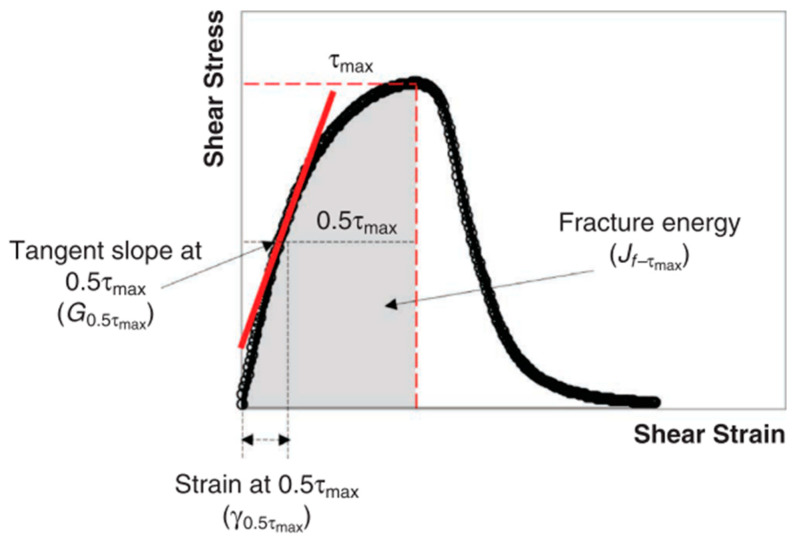
Typical PLAS Test Data and FREI Parameter Definitions. Reprinted with permission from Ref. [24].

The second approach was the Pure Linear Amplitude Sweep (PLAS) method proposed by Zhou et al. [24], which uses the parameter Fatigue Resistance Energy Index (FREI) to compare the binders’ fatigue behaviour, as shown in Figure 6.

FREI can be calculated using the following Equation:

(2) $$\mathrm{FREI}=\frac{J_{f-\tau_{max}}}{G_{0.5_{\tau_{max}\;}}}{\left(\gamma_{0.5}{}_{\tau_{max}}\right)}^{2}$$

where:

$J_{f-\tau_{max}}$: the shear fracture energy at the peak shear stress
$G_{0.5_{\tau_{max}\;}}$: the calculated shear modulus at 0.5τ_max_
$\gamma_{0.5}{}_{\tau_{max}}$: the shear strain at 0.5τ_max_ (different from 0.5γpeak)

Zhou et al. [24] found that the larger the FREI, the better the fatigue cracking resistance. The test was repeated three times, and the average value was used in the analyses.

### 3.3. PLASH Test

**Figure 7:**
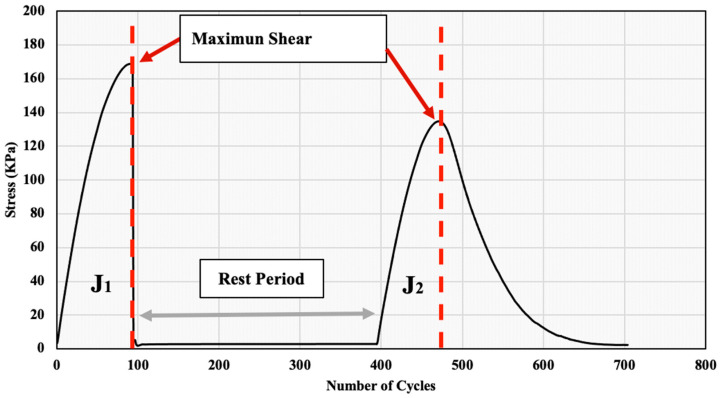
PLAS-Healing Analysis Diagram (J_2_ and J_1_ represent the shear fracture energy calculated until maximum shear stress).

The self-healing ability was evaluated at a temperature of 20 °C using the simplified LASH test for all asphalt samples presented in Table 2. LASH tests were conducted in several studies to investigate the healing capacity of asphalt binders [25]. However, the PLASH test used in this study was developed at The Centre for Pavement and Transportation Technology (CPATT). As shown in Figure 7, an oscillatory strain sweep was applied to the asphalt samples, with strain amplitudes linearly ranging from 0.1% to a specified strain level. This strain level is associated with the peak shear stress found in the PLAS test.

Furthermore, the test involved introducing a rest period, after which another loading phase was introduced, ranging from 0.1% to 30% at 10 Hz at the same temperature. It is worth mentioning that six samples were tested for each binder type. To investigate the effect of the rest period on the healing capacity, three samples were subjected to a 5 min rest period. However, the rest of the samples were subjected to a 30 min rest period.

The PLASH method uses the parameter FREI to evaluate the fatigue cracking resistance of the asphalt binder before and after the rest period. The Rest index (%Res) uses only fracture energy to evaluate the healing characteristics, as given in the following Equation:

(3) $$\%\mathrm{Res}=\frac{J_{2}}{J_{1}}$$

where: *J*_2_ and *J*_1_ represent the shear fracture energy calculated until maximum shear stress. The higher the %Res, the higher the healing capacity.

## 4. Results and Discussion

### 4.1. LAS Results

**Figure 8:**
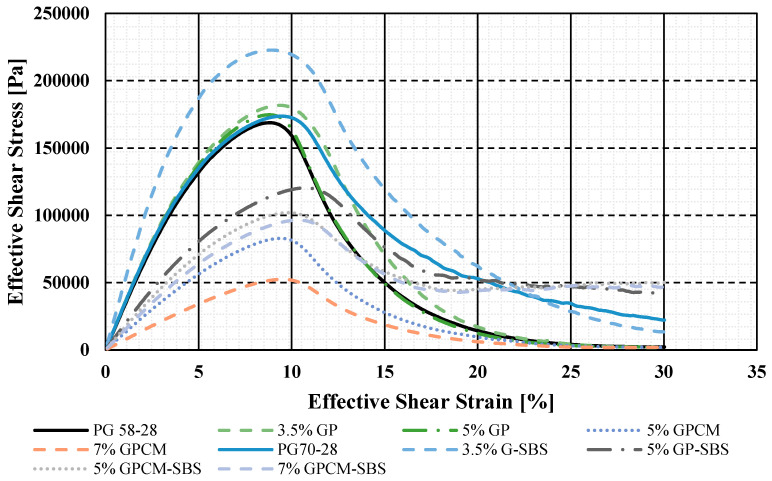
Shear Stress versus Shear Strain (LAS Test).

The results of the shear stress evolution for all the tested binders during the LAS test are illustrated in Figure 8. The data for both axes are plotted on normal scales of effective shear stress (Y-axis) versus effective shear strain (X-axis). The test is used to characterise the different binders’ fatigue behaviour using the LAS procedure [26]. The figure shows that 7%GPCM, 5%GPCM, 5%G-SBS and 3.5%G-SBS binders tend to result in curves with more broadened peaks compared to the PG 58-28 binder. Moreover, there was a noticeable increase in the strain values corresponding to the maximum stress levels of the modified binders with GP and GPCM. The highest strain values were obtained for 3.5%GP-SBS and 5%GP-SBS, as shown in the figure, whereas the PG 58-28 binder’s strain value corresponding to the maximum stress levels was 8.75%. Therefore, it can be concluded that the fatigue life values of the 7%GPCM, 5%GPCM, 5%GP-SBS and 7%GPCM-SBS binders are expected to increase.

### 4.2. Fatigue Life Prediction

**Figure 9:**
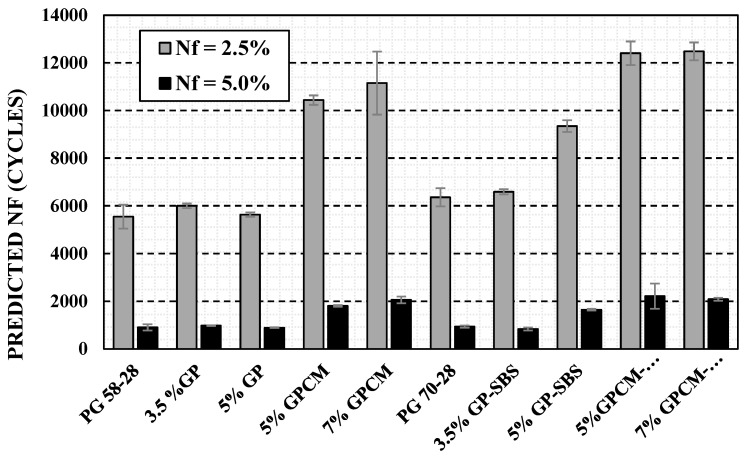
Predicted Fatigue Life Values at 2.5% and 5.0% Strain Amplitude Using VECD Approach.

Figure 9 shows the predicted fatigue life for all binders at 2.5% and 5.0% strain amplitudes (N_f_) for all tested binders proposed in the VECD approach. The predicted fatigue life of PG 58-28 (N_f_) was 5546, while those of the mixes with GP were 5999 and 5635 for 3.5%GP and 5%GP, respectively. The two GPs’ N_f_ values were quite close to the one of the PG 58-28 binder, indicating that the use of GP alone was not useful in terms of fatigue. When PCM and SBS are used, the fatigue life showed a significant improvement, as anticipated from the LAS test results. The predicted fatigue life values for 7%GPCM-SBS, 5%GPCM-SBS, 7%GPCM and 5%GPCM at 2.5% strain amplitude were 12,482, 12,403, 11,151 and 10,436, respectively. As expected from the shear stress versus shear strain curves presented in Figure 9, the addition of GPCM modifiers has significantly increased the fatigue life values for the binders.

The fatigue life improvement in the case of the GPCM would be explained by the fact that these binders act like a mastic rather than a binder. The friction that the GP particles create in the binder is most likely increasing the stress needed to deform the binder, which would be the reason for the decrease in the damage rate and the increase in the fatigue life. Therefore, it would be inaccurate to conclude that the use of GPCM improves fatigue life without conducting fatigue tests at the mix level.

### 4.3. Simplified Viscoelastic Continuum Damage Modeling (S-VECD)

**Figure 10:**
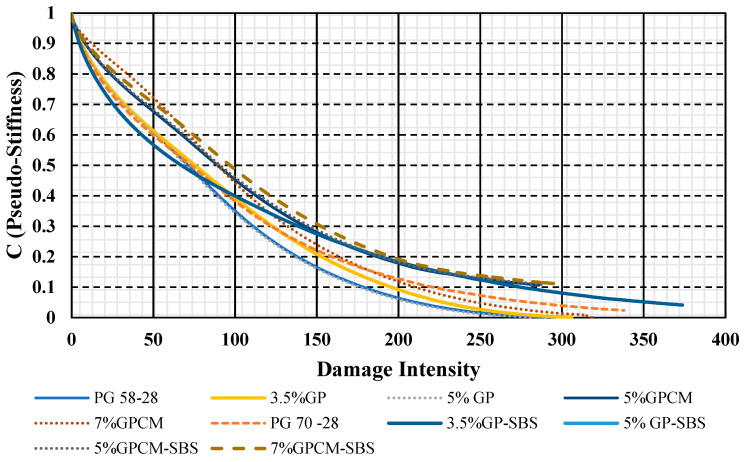
VECD Damage Characteristics: Pseudo-Stiffness Versus Damage Parameter.

The viscoelastic continuum damage model was used to predict the fatigue life of asphalt binders with different additives. The S-VECD damage characteristic curves are shown in Figure 10. The figure shows the material stiffness changes at different strain levels. In other words, it shows the relationship between the material modulus, pseudo-stiffness (C) and its damage parameter (S). It can be noticed that the damage evolution curves of the 3.5%GP and 5%GP binders had a similar trend to that of the PG 58-28 binder.

Moreover, the PG 70-28 binder showed an overall lower damage evolution during the test, which can be contributed to the elastic and high stiffness properties that the SBS possesses. For PG 58-28, PG 70-28, 3.5%GP and 5%GP, the damage curves showed a steeper trend at the beginning of the loading phase at a damage intensity of less than 50 compared to the rest of the binders. Additionally, these binders showed lower damage evolution than the PG 58-28 binder.

**Figure 11:**
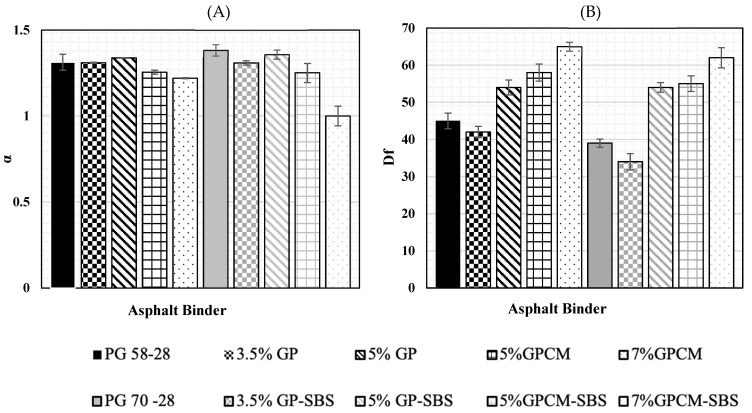
Effect of Different Additives on the Damage Performance: (**A**) a Parameter (**B**) Damage at Failure (D_f_).

In addition, the effect of different additives on the damage performance in terms of α parameter and (D_f_) damage at failure is shown in Figure 11. The α parameter represents the damage evolution rate, and (D_f_) represents the value of D(t) when the material integrity decreased to 65% of its initial value [23]. An indication of a desirable fatigue resistance performance can be achieved by obtaining lower and higher values of α and D_f_, respectively [23]. As can be seen, the 7%GPCM binder had the highest capacity to accumulate damage before failure (up to 62%) and had a relatively lower damage evolution rate α of 1.22. On the contrary, the PG 58-28 and PG 70-28 binders had the lowest capacity to accumulate damage, with values of 39% and 45%, respectively, before failure and damage evolution rates α of 1.383 and 1.313, respectively

### 4.4. Asphalt Binder Ranking Based on LAS Analysis

Based on the LAS analysis, a relative ranking table was developed and presented in Table 3. The ranking criteria are based on the predicted fatigue life at a 2.5% strain level. The rankings range from 1 to 10, where #1 represents the best fatigue life, and #10 represents the lowest fatigue life among the tested binders.

According to the analysis, the 7%GPCM binder showed the highest predicted fatigue life. However, the addition of 3.5% and 5%GP did not result in a noticeable improvement in fatigue performance. This is primarily because GP would act as a filler to increase the viscosity of the binder. It was mentioned earlier that the aim of adding GP to the binders was to improve the stability of the mastic and compensate for the loss of stiffness caused by the PCM.

### 4.5. PLAS Results

**Figure 12:**
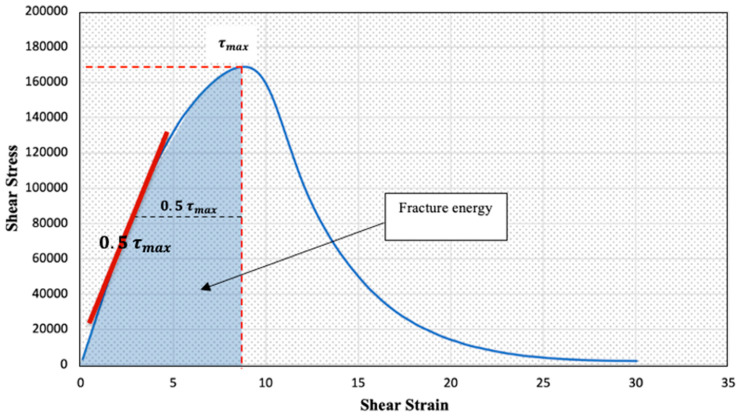
PLAS Test Data and FREI Parameter Values for the PG 58-28 Binder.

The stress-strain curves from the PLAS tests for all the tested asphalt binders are presented in Figure 8. The curves were used to obtain the maximum shear stress values (τ_max_) for each binder. Then, these values were used to calculate the fracture parameters (FREI) using Equation (2). For example, Table 4 and Figure 12 show the calculation procedures for the PG 58-28 binder.

**Figure 13:**
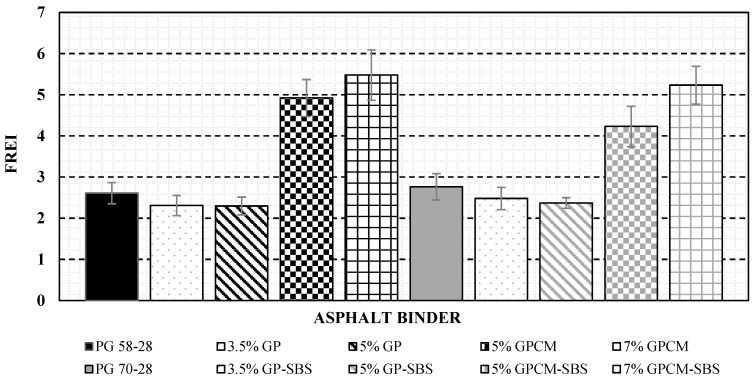
PLAS Test Results (FREI) for Different Additives.

Figure 13 shows the PLAS test results (FREI) for all tested asphalt binders. The higher the FREI values, the better the fatigue resistance. Similar to the LAS analysis, binders with GPCM exhibited better fatigue resistance when analysed using the PLAS method. The results showed that binders modified with GPCM had the highest values of FREI (above 4.2). On the other hand, binders modified with GP showed the lowest fatigue resistance with the lowest FREI values among the tested binders. The reduction in the FREI values of modified binders with GP can also be attributed to the high stiffness of these binders.

### 4.6. Asphalt Binder Ranking Based on PLAS Analysis

Similar to the LAS analysis, a ranking table was developed for all the tested binders under the PLAS test, as shown in Table 5. The ranking was conducted based on FREI values. The rankings range from 1 to 10, where #1 represents the best fatigue resistance (highest FREI value), whereas #10 represents the lowest fatigue resistance (lowest FREI value) among the tested binders. The highest FREI value was calculated for the 7%GPCM binder at a value of 5.47, whereas the lowest was recorded for the 5%GP binder at 2.29. This indicates that the 7%GPCM binder would show the highest fatigue resistance, while the 5%GP binder would be the least performant binder in terms of fatigue.

Generally, even though two methods were adopted to rank the tested asphalt binders, LAS and PLAS methods, it was observed that the additions of both 5% and 7%GPCM to the asphalt binder improved the fatigue performance based on both methods. Moreover, the additions of 3.5% and 5%GP to the PG 58-28 binders were found to be at the lower tail of the ranking in terms of fatigue resistance for both methods.

### 4.7. PLASH Results

The fatigue test discussed in the previous section was performed without introducing a rest period. Therefore, to investigate the self-healing ability of asphalt binders, a rest period was introduced during the LAS test. The results obtained from the previous sections were used to identify the maximum shear stresses, for all the tested binders, at which the rest period was introduced for either 5 or 30 mins.

**Figure 14:**
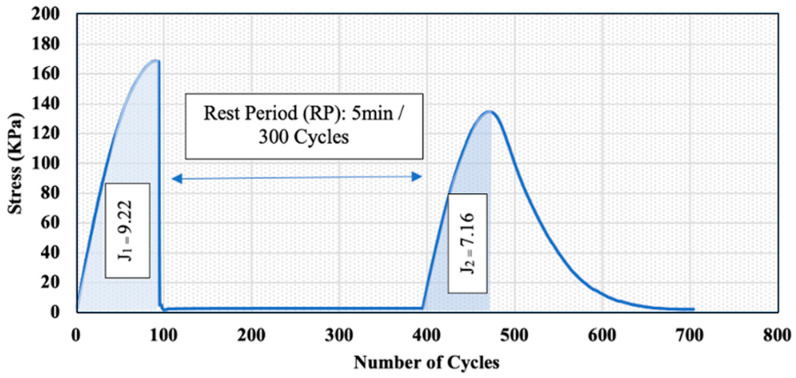
Sample Curve for the PG 58-28 Binder and the Parameters Used to Calculate the Rest Index.

The PG 58-28 binder was used as an example to show the calculation procedure and all the parameters for the 5 min rest period to calculate the Rest index (%Res), as shown in Figure 14. J1 and J2 were calculated as 9.22 and 7.16, respectively. These values were obtained by calculating the area under the curve till the max shear stress (τmax). The Rest index (%Res) was calculated using Equation (3) and found to be 77.67%. In other words, the total observed restoration after the rest period was 77.67% of the binder’s initial fracture energy.

**Figure 15:**
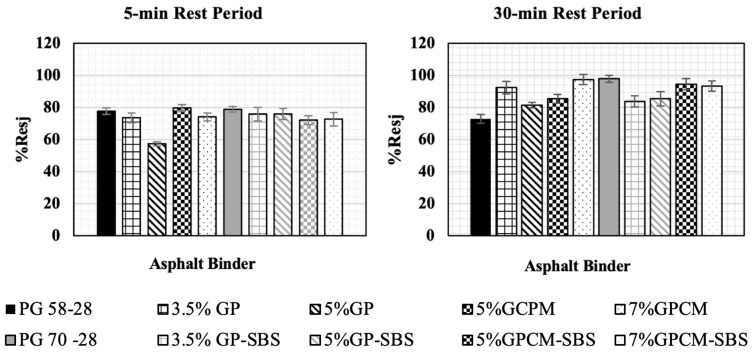
Rest Index (%Res) Values Calculated for all Binders.

Moreover, the %Res values for all binders at 5 and 30 min rest periods are presented in Figure 15. For the 5 min rest period, the %Res values were lower for almost all modified binders compared to that of the PG 58-28 binder (less than 77.67%). The %Res for the 5%GP value was recorded at 57.26%, which is the lowest healing capacity among all the tested binders. Additionally, the %Res values for the 5%GPCM and PG 70-28 binders were found to be 79.5% and 78.9%, respectively, slightly higher than that of the PG 58-28 binder, as illustrated in Figure 15. The slight increase can be explained as the result of the cross-linking segments of the SBS chain in the asphalt structure, which led to an improvement in the healing during the rest period. This improvement could be explained by the softening effect of the GPCM, which could have accelerated the flow and the wetting during the healing process. On the other hand, when a 30 min rest period was introduced, all binders showed an improvement in the healing capacity by over 75% compared to the PG 58-28 binder. These findings are in agreement with the study reported by [3], in which the rest period duration had a significant impact on the healing capacity of the asphalt binder.

As shown in Figure 15, the highest healing capacity was obtained for the PG 70-28 binder at 97.9%. This can be attributed to the presence of SBS in the binder, which has a rubbery nature with high chain connectivity. These properties contributed to the chain’s high strength and the flexible behaviour of the binder, which led to the increased recovery capacity of the asphalt binder during the rest period. However, the additions of 3.5% and 5%GP to the PG 70-28 binder resulted in reductions of the healing capacity by approximately 14.5% and 12.4%, respectively. This might be due to the fact that the addition of GP tended to increase the brittleness of the binder. Additionally, the highest %Res values of 97.4%, 94.58% and 93.32% were obtained for the 7%GPCM, 5%GPCM-SBS and 7%GPCM-SBS binders, respectively, which indicated that the binder restoration capacity reached almost its initial integrity. An interesting finding was observed for the PG 58-28 binder: unlike with the 5 min rest period, when the 30 min rest period was introduced, the %Res value was the lowest among all the tested binders.

### 4.8. Asphalt Binder Ranking Based on PLASH Analysis

Two rest periods with different durations of 5 and 30 mins were introduced at the maximum shear stress of the PLASH tests for each binder. The %Res values were calculated and displayed in Figure 15. These values were used to create a ranking table for all the tested binders, as shown in Table 6. The rankings range from 1 to 10, where #1 represents the highest healing capacity, and #10 represents the lowest healing capacity among the tested binders.

### 4.9. Radar Chart

**Figure 16:**
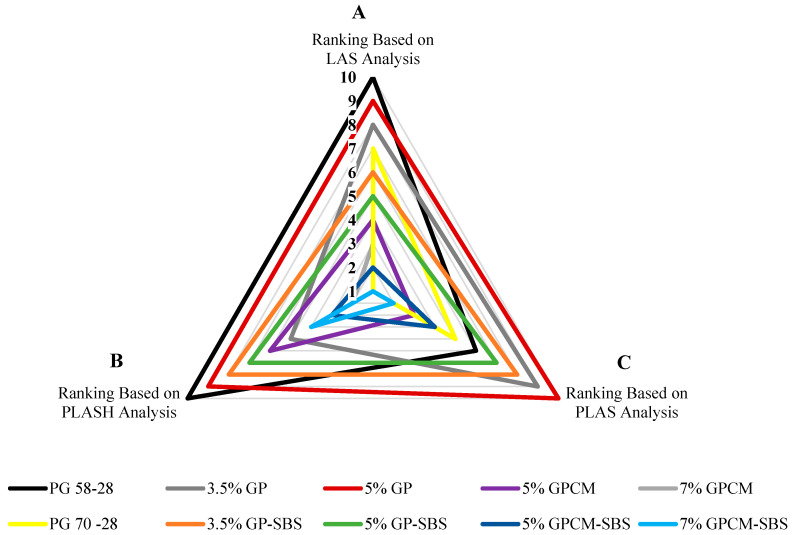
Rankings of all Binders based on (A) LAS, (C) PLAS and (B) PLASH Analysis. #1 represents the highest performance, and #10 represents the lowest performance among the tested binders.

To better understand and visualise the ranking tables, a radar chart was used to summarise the obtained results from this study, as shown in Figure 16. The chart shows a multivariate ranking approach for each binder in the form of a two-dimensional chart. The chart displays the obtained results for each analysis of the tested binders (LAS, PLAS and PLASH), along with their ranking.

Three notations (A, B and C) are used to represent the ranking of the binders, based on the test analysis used on the chart. Binder rankings based on the LAS analysis are represented by the notation (A); however, notations B and C are used to represent the binder rankings based on the PLASH (at 30mins rest period) and PLAS analyses, respectively.

As can be seen from Figure 16, the figure consists of different triangles, and each triangle represents one type of binder; for example, the green triangle represents the 5%GP-SBS binder. It is clearly shown that as the area of the triangle becomes smaller, the the binder performance becomes better. For instance, the smallest triangle area was obtained for the 7%GPCM binder (gray triangle), indicating that the 7%GPCM binder exhibited the best performance (Fatigue and Self-healing) among all the binders. However, the largest area among all the triangles was calculated for the 5%GP binder (red triangle), indicating its poor performance in terms of fatigue and self-healing when analysed using the three different methods.

Moreover, it can be noted that although analysing the PG70-28 binder (orange triangle) using the PLASH method exhibited the best healing capacity (Rank #1), its fatigue behaviour was not as expected as the healing behaviour, where it was ranked as #6 and #7 based on PLAS and LAS analysis, respectively.

### 4.10. ANOVA Analysis

The influence of the length of the rest period was investigated by performing a two-way analysis of variance (ANOVA) analysis to evaluate the effect of all the additives (GP, GPCM, SBS-GP and SBS-GPCM) at different percentages (3.5%, 5% and 7%) and rest periods (times) on the healing capacity. Table 7 shows the obtained results from the ANOVA analysis for the different mixes. It is clearly shown that the rest period has a significant effect on the healing capacity for all mixes since the *p*-values for all mixes are less than 0.005. Moreover, ANOVA analysis showed that the modification rate significantly affected the healing capacity for all the mixes based on *p*-values presented in Table 7.

## 5. Conclusions

A total of 90 specimens were prepared and tested to evaluate the self-healing and fatigue resistance properties of asphalt binders using DSR. Sixty and thirty specimens were prepared to evaluate the self-healing properties and the fatigue resistance, respectively.

The tested binders, PG 58-28 and PG 70-28, were modified with two types of additives, GP and GPCM. The GP was added in the form of powder, whereas the GPCM was added in a paste form, both of which were added at different percentages of 3.5%, 5% and 7% by binder weight.

Two different procedures, one with a rest period and another without a rest period, were followed to conduct the LAS test. A rest period of 5 or 30 min was introduced to the LAS test, and the healing index was measured. Three categories were used to classify the tested binders based on their self-healing capacity and fatigue performance. The Linear Amplitude Sweep (LAS), Pure Linear Amplitude Sweep (PLAS) and modified Pure Linear Amplitude Sweep Healing (PLASH) were used to rank the binders based on their performance. Some conclusions were drawn:

- Based on the LAS analysis, at a 2.5% strain level, PG 58-28 has poor fatigue resistance compared to the other tested binders. In contrast, the 7% GPCM binder addition showed the best fatigue resistance among all tested binders.
- The 7%GPCM binder showed the highest capacity to accumulate damage (Df) before failure and a relatively lower damage evolution rate α. Similarly, the same conclusions were drawn when the data were analysed using the PLAS method.
- It was found that the calculated FREI values obtained for binders modified with GPCM ranged from (4.2 to 5.4). As for the modified binders with GP, the calculated FREI values and measured fatigue resistance were the lowest among the tested binders.
- The additions of 5% and 7%GPCM resulted in good fatigue resistance. Still, they resulted in a minimal improvement in the self-healing capacity of the binders when the rest period was limited to 5 mins.
- When a 5 min rest period was introduced, the PG70-28 binder exhibited the best healing capacity, with a %Res of 79.5%.
- Unlike the 5 min rest period, when a 30 min rest period was introduced, the %Res increased noticeably for all the modified binders compared to that of the base binders, particularly binders modified with GPCM.
- Two-way ANOVA analysis showed a significant impact of the rest period durations (5 and 30 mins) on the healing capacity. The longer the rest periods, the better the healing behaviour of the asphalt binders.

## Figures and Tables

**Table 1:** Properties of the PG 58-28 and PG 70-28 Asphalt Binders. Table reprinted from Ref. [13] with permission of Company Yellowline Asphalt Products.

**Properties of PG 58-28 Binder**					
Index	Conditions (°C)	Unit	Results	Requirements	Test Method
Specific gravity	At 15		1.03	-	
Brookfield viscosity	At 135	Pa∙s	0.275	3.0 max	AASTHO T316
Flash point	-	°C	230+	230 min	AASHTO T 48
G*/sin(δ)	At 58	kPa	1.195	5 min	AASTHO T315
**Properties of PG 70-28 Binder**					
Index	Conditions (°C)	Unit	Results	Requirements	
Specific gravity	At 25		1.03	-	
Brookfield viscosity	At 135	Pa∙s	0.9	3.0 max	AASTHO T316
Flash point	-	°C	230+	230 min	AASHTO T 48
G*/sin(δ)	At 58	kPa	3.64	1.0 min	AASTHO T315

**Table 2:** Nomenclature for Asphalt Binders.

Binder Identification	Base Binder	Additives
PG 58-28	PG 58-28	-
PG 70-28	PG 58-28	3% Styrene-butadiene-styrene
5% GPCM	PG 58-28	5% (Glass Powder + Phase Chane Materials)
7% GPCM	PG 58-28	7% (Glass Powder + Phase Chane Materials)
5% GPCM-SBS	3% SBS modified PG 58-28	5% (Glass Powder + Phase Chane Materials)
7% GPCM-SBS	3% SBS modified PG 58-28	7% (Glass Powder + Phase Chane Materials)
3.5% GP	PG 58-28	3.5% (Glass Powder)
5% GP	PG 58-28	5% (Glass Powder)
3.5% GP-SBS	3% SBS modified PG 58-28	3.5% (Glass Powder)
5% GP-SBS	3% SBS modified PG 58-28	5% (Glass Powder)

**Table 3:** Rankings of Asphalt Binders Based on Fatigue Performance (LAS Analysis).

Binder Rank	Based on Nf at 2.5%	Nf at 2.5%
1	7% GPCM-SBS	12,482
2	5% GPCM-SBS	12,403
3	7% GPCM	11,151
4	5% GPCM	10,436
5	5% GP-SBS	9346
6	3.5% GP-SBS	6590
7	PG 70-28	6358
8	3.5% GP	5999
9	5% GP	5635
10	PG 58-28	5546

**Table 4:** Sample Calculations of FREI for the PG 58-28 Binder.

Parameter	Value	Unit
τmax	168	kPa
Nτ-max	881	cycles
Jf-τmax	9.31	kPa
N0.5τ-max	280	cycles
G0.5τ-max	2640	kPa
γ0.5τ-max	2.720	%
$FREI=\frac{J_{f-\tau_{max}}}{G_{0.5_{\tau_{max}\;}}}{\left(\gamma_{0.5}{}_{\tau_{max}}\right)}^{2}$	2.610	

**Table 5:** Rankings of Asphalt Binders Based on Fatigue Performance (PLAS Analysis).

Binder Rank	Based on FREI	FREI Values
1	7%GPCM	5.47
2	7%GPCM-SBS	5.23
3	5%GPCM	4.92
4	5%GPCM-SBS	4.23
5	PG 70-28	2.76
6	PG 58-28	2.61
7	5% GP-SBS	2.53
8	3.5% GP-SBS	2.48
9	3.5% GP	2.31
10	5% GP	2.29

**Table 6:** Rankings of Asphalt Binders Based on Self-Healing Capacity (PLAH).

Binder Rank	5 min Rest Period	30 min Rest Period
1	5% GPCM	PG 70-28
2	PG 70-28	7%GPCM
3	PG 58-28	5%GPCM-SBS
4	3.5% GP-SBS	7%GPCM-SBS
5	5% GP-SBS	3.5% GP
6	7% GPCM	5%GPCM
7	3.5% GP	5% GP-SBS
8	7% GPCM-SBS	3.5% GP-SBS
9	5% GPCM-SBS	5% GP
10	5% GP	PG 58-28

**Table 7:** Two-way analysis of variance (ANOVA) analysis for each additive.

**GPCM**	
**Source of Variation**	*p*-value
**Time**	0.00676682
**Additives**	0.00498868
**GP**	
**Source of Variation**	*p*-value
**Time**	0.00013471
**Additives**	0.23194346
**SBS-GP**	
**Source of Variation**	*p*-value
**Time**	0.00359623
**Additives**	0.10570633
**SBS-GPCM**	
**Source of Variation**	*p*-value
**Time**	4.5173 × 10^−7^
**Additives**	0.10184976

## Data Availability

The data that support the findings of this study are available from the first author, HA, upon reasonable request.
